# Multiple Modalities of Sensory Gating Are Affected in Decompensated Tinnitus

**DOI:** 10.1097/AUD.0000000000001838

**Published:** 2026-06-01

**Authors:** Jana V. P. Devos, Pia Brinkmann, Petra Vavami, Marcus L. F. Janssen, Michael Schwartze, Sonja A. Kotz

**Affiliations:** 1 Mental Health and Neuroscience Research Institute, Maastricht University, Maastricht, the Netherlands; 2 Department of Ear, Nose, Throat, Head and Neck Surgery, Maastricht University Medical Center, Maastricht University, Maastricht, the Netherlands; 3 Department of Neuropsychology and Psychofarmaology, Faculty of Psychology and Neuroscience, Maastricht University, the Netherlands; 4 Department of Clinical Neurophysiology, Maastricht University Medical Center, Maastricht University, Maastricht, the Netherlands.

**Keywords:** Questionnaire study, Sensory Gating, Sensory Gating Inventory, Tinnitus, Tinnitus Questionnaire

## Abstract

**Objectives:**

Sensory gating (SG) is the ability to differentiate novel or salient sensory input from known or irrelevant information. In tinnitus, SG is considered dysfunctional, as the brain fails to inhibit internal auditory neural activity before it reaches conscious perception. As a result, the brain continuously processes the tinnitus signal as if it was a new and relevant stimulus. SG can be assessed with a self-report tool, the Sensory Gating Inventory (SGI).

**Design:**

A prospective questionnaire study was performed in persons with tinnitus (n = 358) and a control group without tinnitus (n = 436). Tinnitus severity was assessed with the tinnitus questionnaire (TQ) and used to split the tinnitus group into subgroups of persons with compensated and decompensated tinnitus. The decompensated tinnitus subgroup consisted of persons suffering from severe tinnitus, while the compensated subgroup consisted of persons with less severe tinnitus. Systematic covariation measures were applied to assess the association between the TQ and the SGI results.

**Results:**

SG was impaired in the domains of Perceptual Modulation, Distractibility, and Vulnerability to Fatigue and Stress as well as in the auditory modality in persons with tinnitus when compared with persons without tinnitus. When the tinnitus group was divided into the subgroups, higher SGI scores on all subscales were prevalent in the decompensated tinnitus subgroup. The results in the compensated tinnitus subgroup were more similar to the control group, except for the auditory modality.

**Conclusions:**

The decompensated tinnitus subgroup showed higher scores on all SGI subscales, which might indicate a more substantial SG impairment. The relation between TQ and the SGI results and the subgrouping of persons with tinnitus can consequently inform and improve future research about the transitional period and the processes that dysregulate when compensated tinnitus turns into decompensated tinnitus.

## INTRODUCTION

Tinnitus can be defined as the perception of a sound in the absence of an external sound source. Up to 14.7% of the general population experience tinnitus, of which 1.2% are severely impacted in their daily life activities (Jarach et al. 2022).

To function efficiently in daily life, it is essential to identify and filter irrelevant sensory information. The putative underlying automatic filtering mechanism is called sensory gating (SG) (Adler et al. 1982). SG is defined as the preattentive ability to differentiate novel or salient sensory input from known or irrelevant information. This filtering essentially reduces processing costs at higher cortical brain areas, such as the auditory cortex, and improves responsiveness when stimulation changes (Cromwell et al. 2008). This is an important protective mechanism that ensures the brain is not constantly overwhelmed by redundant sensory information and which allows focus on the most pertinent stimuli. A tinnitus sound is uninformative and usually constant. It would therefore be considered an irrelevant stimulus.

Persons experiencing tinnitus can be divided into two groups, namely compensated and decompensated tinnitus (Goebel & Hiller 1994; Lenarz 1998). Persons with compensated tinnitus rarely experience burden or consequences on their daily life from the tinnitus. However, tinnitus leads to distress and annoyance in persons with decompensated tinnitus (Stobik et al. 2005). Such negative impact on daily life can present as an interference with work, impaired social interaction, or emotional distress (Tyler & Baker 1983; Folmer et al. 1999; Folmer & Griest 2000). In addition, persons suffering from tinnitus tend to have overall decreased health and are more prone to depression, anxiety, and insomnia (Schleuning 1991; Folmer & Griest 2000; Crocetti et al. 2009; Cho et al. 2013).

A stimulus can gain relevance through emotional valence (positive/negative) and arousal attribution, which provide bodily and experiential information about the value and importance of the encountered stimulus. Valence and arousal associated with a tinnitus sound can therefore bias judgments and decision-making (Storbeck & Clore 2008). When a tinnitus sound acquires emotional significance and elicits arousal, it may become distressing (McKenna et al. 2014). SG is considered dysfunctional in tinnitus, as the brain fails to inhibit the internally generated auditory neural activity before it reaches conscious perception. As a result, the tinnitus signal continues to be processed as if it was a new and relevant stimulus. This mechanism appears to be more strongly affected more in persons with decompensated tinnitus, as shown by the greater impact on daily life.

Neurophysiological aspects of SG can be investigated using cortical auditory evoked potentials that can be quantified with measures of amplitude or latency. SG is often investigated by presenting pairs of identical sound stimuli. Typically, this results in a decrease in amplitude of the response to the second stimulus as compared with the first stimulus; the response to the second stimulus is inhibited. This might indicate the relative success of filtering out irrelevant/repetitive information. In addition, other aspects of SG are reflected in the latency. This is typically observed as increased latency in response to the second stimulus (Morse et al. 2024). In adults with tinnitus, neurophysiological studies investigating SG results were inconclusive concerning the specific neurophysiological measures (Dornhoffer et al. 2006; Campbell et al. 2018; Brinkmann et al. 2024; Morse et al. 2024), which likely reflects the heterogeneity of the condition (Cederroth et al. 2019). In sum, Campbell et al. (2018, 2019) showed that the gating function was impaired in persons with tinnitus and electrophysiological measures could be correlated with tinnitus severity, even in persons with compensated tinnitus (Campbell et al. 2018, 2019). Comparably, Morse et al. (2024) showed that in comparison, the tinnitus group had electrophysiological characteristics consistent with poorer auditory gating (Morse & Vander Werff 2024; Morse et al. 2024).

Behavioral and clinical aspects of SG can be measured by means of self-reports, such as the Sensory Gating Inventory (SGI) (Hetrick et al. 2012). The SGI quantifies multiple dimensions of SG, such as perception, fatigue, and stress, across different modalities (auditory, visual, and attentional modalities). The SGI indicated an SG deficit in schizophrenia (Micoulaud-Franchi & Vion-Dury 2013; Micoulaud-Franchi et al. 2014b), attention-deficit hyperactivity disorder (ADHD) (Sable et al. 2012; Micoulaud-Franchi et al. 2015a, b, 2019), and Tourette syndrome (Sutherland Owens et al. 2011). Moreover, these deficits in SG as measured by the SGI have been correlated with neurophysiological data in both Schizophrenia (Micoulaud-Franchi et al. 2014a) and ADHD (Micoulaud-Franchi et al. 2015b). In addition, in a small Persian-speaking participant group, SGI scores were significantly correlated with tinnitus severity (Mohebbi et al. 2019a, b). This finding has so far not been replicated, although a self-report measure of SG would be of high interest for tinnitus research in general and for studies of tinnitus across languages in particular. The aim of the present study was therefore to systematically investigate the relationship between tinnitus severity and significant changes in SG in a Dutch-speaking population. We expected similar SG between the compensated tinnitus group and a healthy control group, while we predicted the decompensated tinnitus group to express impaired SG. We further expected a significant difference between all three groups for SG in the auditory modality. For the other modalities (visual and focus), we did not expect the groups to differ as tinnitus is a deficit in the auditory modality.

## MATERIALS AND METHODS

### Participants

Participants were recruited via an existing database at NAME MEDICAL CENTER that includes persons with tinnitus who indicated willingness to take part in research. In addition, a flyer was shared through the social media channels of the Dutch patient association (Stichting Hoormij).

### Procedure

An online survey was designed using the Qualtrics (Provo, UT) platform. It consisted of the Dutch SGI (SGI-D; Hetrick et al. 2012; Brinkmann et al. 2023), the Dutch Tinnitus Questionnaire (TQ) (Goebel & Hiller 1994; Meeus et al. 2007), and questions on demographics and tinnitus characteristics (Table 1) taken from the ESIT-SQ (Genitsaridi et al. 2019).

**TABLE 1.** Characteristics of the tinnitus participants, count, and percentage given unless indicated differently

Variable	n (%)		
	Compensated Tinnitus	Decompensated Tinnitus	Total Tinnitus
Tinnitus diagnosed by physician	225 (87)	91 (89)	316 (88)
Comorbid hearing loss	100 (39)	51 (20)	151 (42)
Type of tinnitus sound			
Combinations of sounds	125 (49)	59 (58)	184 (51)
Tonal-like	74 (29)	26 (25)	100 (28)
Noise-like	57 (22)	18 (18)	75 (21)
Other	13 (5)	5 (5)	18 (5)
Crickets	6 (2)	1 (1)	7 (2)
Tinnitus location			
Bilateral	147 (57)	61 (60)	208 (58)
Lateralized right	47 (18)	23 (23)	70 (20)
Lateralized left	50 (20)	22 (22)	72 (20)
Equal	50 (20)	16 (16)	66 (18)
Unilateral	61 (23)	17 (17)	78 (22)
Left	34 (13)	11 (11)	45 (13)
Right	27 (11)	6 (6)	33 (9)
Inside head	38 (15)	17 (17)	55 (15)
Other	9 (4)	7 (7)	16 (5)
TQ Grades			
1	139 (54)		
2	117 (33)		
3		61 (17)	
4		41 (11)	
TQ total score (M, SD)	28 (11)	58 (9)	37 (17)
Tinnitus duration (y) (M, SD)	11 (11)	8 (7)	10 (10)
VAS Tinnitus loudness 0–100 (M, SD)	63 (21)	79 (12)	68 (20)

TQ, tinnitus questionnaire; VAS, Visuals Analog Scale.

### Sensory Gating Inventory

SG was assessed using the SGI-D (Brinkmann et al. 2023), which is based on the English SGI by Hetrick et al. (2012). This 36-item self-report measure uses a 6-point Likert scale, ranging from “never true” (0) to “always true” (5). Persons who score high on the SGI are likely to have difficulty inhibiting irrelevant sensory information. The SGI consists of four subscales: Perceptual Modulation (PM; 16 items), Distractibility (D; 8 items), Over-Inclusion (OI; 7 items), and Vulnerability to Fatigue and Stress (FS; 5 items). PM evaluates an individual’s subjective experience of the ability to perceptually modulate and filter sensory stimuli, often described as the experience of being flooded with sensory stimuli. OI evaluates the tendency to over-include or over-attend to extraneous sensory stimuli. D concerns the susceptibility to distraction by other stimuli. FS investigates the vulnerability of the perceptual processes to fatigue and stress (Bailey et al. 2021).

The SGI covers several modalities: auditory (17 questions concerning auditory perception [9 PM, 3 D, 3 OI, 2 FS] scores ranging 0 to 85), visual (10 questions concerning visual perception [5 PM, 0 D, 2 OI, 3 FS] scores ranging 0 to 50), 2 questions that concern both the auditory and visual modality (both PM), and 7 questions about the ability to focus their attention (concerning sustained attention [0 PM, 5 D, 2 OI, 0 FS] and switching focus scores ranging 0 to 30).

### Tinnitus Questionnaire

Tinnitus severity was assessed with the Dutch version of the TQ (Goebel & Hiller 1994; Meeus et al. 2007). The TQ consists of 52 items scored on a 3-point Likert scale (2 “correct,” 1 “partly correct,” and 0 “incorrect”) with 7 reverse-phrased items. The total TQ score can range from 0 to 84 and be classified into 4 grades for clinical purposes, with compensated tinnitus: grade 1, little distress (0 to 30); grade 2: medium distress (31 to 46) and decompensated tinnitus; grade 3: severe distress (47 to 58); and grade 4: very severe distress (59 and up). A decrease of at least 10 points means a significant change in Tinnitus severity (Adamchic et al. 2012).

### Statistical Analyses

The data were analyzed using IBM SPSS Statistics version 27.0 (IBM Corp., Armonk, NY). All variables are described using mean (M) and SD.

A χ^2^ test was used to compare sex differences between the control group and the tinnitus group. An independent *t* test was used to compare age differences between the control group and the tinnitus group.

As it was previously shown that both age (Cheng et al. 2015; Pesonen et al. 2023) and sex (Tomasi et al. 2008) can impact SG, a linear regression analysis was performed to check for the potential confounding influence of age and sex.

To compare the SGI scores of the different groups (i.e., no tinnitus, compensated tinnitus, and decompensated tinnitus), an analysis of variance was used, correcting for multiple comparisons using Bonferroni adjustment (CDC 2015).

A linear regression model was applied to test the relationship between SGI scores and TQ scores. The variables sex, age, tinnitus loudness, and tinnitus duration were added as proposed in recent tinnitus research (Biswas et al. 2019, 2022; Stohler et al. 2019). With this set of variables, a stepwise backward linear regression analysis was performed, eliminating each variable that was not significant (*p* < 0.05).

Finally, Pearson’s correlations were used to assess covariation between continuous variables; the *p* values were corrected for multiple comparisons using Bonferroni adjustment (CDC 2015). For all statistical analyses, *α* was set at 0.05.

## RESULTS

### Demographics

In total, 795 participants (no tinnitus group n = 436; tinnitus group n = 358) filled out the SGI-D. The control group was significantly younger (M = 30 y, SD = 12 y; *t*(793) = −29.805, *p* < 0.001) and included significantly more (*X^2^* (1, N = 795) = 28.536, *p* > 0.001) females (n = 321, 73.7%) than the tinnitus group (females n = 199, 55.6%) (Table 2). The subsequent group comparisons were therefore controlled for sex and age. The linear regression on the SGI scores showed no influence of age (*B* = 0.06; 95% CI, −0.324 to 0.445; *p* = 0.756) (as could be expected from excluding people older than 65), but it did show an influence of sex (*B* = −16.67; 95% CI, −24.407 to −8.941; *p* < 0.001) with females scoring higher.

**TABLE 2.** Demographics of the population, count, and percentages

Variable	Control Group (n = 436)	Compensated Tinnitus (n = 256)	Decompensated Tinnitus (n = 102)	Tinnitus Total (n = 358)
Men (n, %)	115 (26)	110 (43)	49 (48)	159 (44)
Age (y) (M, SD, range)	30 (SD = 12) range: 19–66	53 (SD = 10) range: 22–65	54 (SD = 10) range: 20–65	54 (SD = 10) range: 20–65

We recorded 424 full responses from persons with tinnitus. Some responses were excluded due to incorrect replies to control questions (n = 6), comorbid disorders that could influence SG (brain damage, current or past substance abuse or dependency, dyslexia, schizophrenia, ADHD/attention-deficit disorder or autism spectrum disorder (n = 14), short duration of experienced tinnitus (e.g., experiencing tinnitus less than once a month) (n = 11), and being older than 65 y (n = 35). Three hundred and fifty-eight responses were thus included in the final analyses. From the validation study for the SGI-D study (Brinkmann et al. 2023), we received 436 responses from participants with no tinnitus diagnosis, which formed the control group.

Next, we took a closer look at the characteristics of the tinnitus group, describing their tinnitus, hearing capacity, and tinnitus severity (Table 1). In the tinnitus group, most participants (n = 316, 88%) reported that their tinnitus was diagnosed by a physician. About 42% of the participants (n = 151) reported comorbid hearing loss (yes/no question). Most participants (n = 167, 47%) reported hearing a combination of sounds or a tone (n = 114, 47%). The majority of participants reported bilateral tinnitus (n = 208, 58%) as opposed to unilateral tinnitus (n = 78, 22%). The mean TQ score for this study population was 37 (SD = 17).

### Comparison Control Versus Tinnitus

The tinnitus group had significantly higher SGI scores than the control group (*B* = 22.771, *p* < 0.001) when controlling for sex and age. This was the case for all subscales (Table 3).

**TABLE 3.** Mean and SD of the SGI and its subscales for the control group and the tinnitus group

Variable	Control Group M (SD)	Tinnitus Group* M (SD)	*p*
SGI Total Score	52 (30)	72 (38)	<0.001
Perceptual Modulation	18 (14)	30 (18)	<0.001
Distractibility	14 (9)	19 (11)	<0.001
Overinclusion	10 (7)	12 (8)	0.008
Fatigue-Stress Vulnerability	10 (6)	12 (7)	<0.001

Differences tested with a regression model accounted for age and sex and were corrected for multiple comparisons using Bonferroni correction.

* Tinnitus group = compensated and decompensated combined.

SGI, Sensory Gating Inventory.

**Fig. 1.**
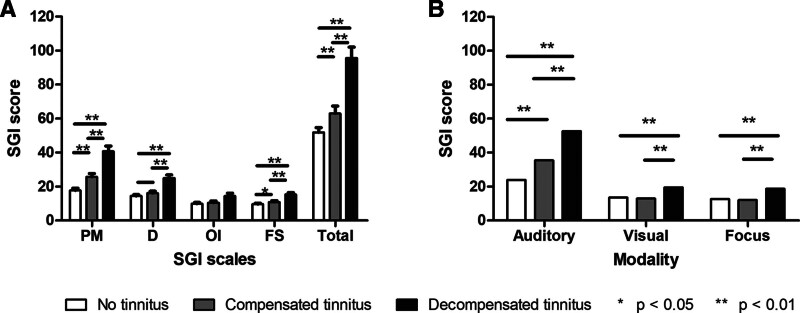
Comparison of SGI subscales between groups. A, Multiple comparisons (Bonferroni corrected) show differences in SGI scores among the studied groups. B, Multiple comparisons (Bonferroni corrected) indicating differences in auditory perception, visual perception, and focus between the studied groups. D indicates Distractibility; FS, Vulnerability to Fatigue and Stress; OI, Over-Inclusion; PM, Perceptual Modulation; SGI, Sensory Gating Inventory.

To differentiate those persons with tinnitus who experienced less distress (compensated tinnitus; TQ score <47) from those with high distress (decompensated tinnitus; TQ score ≥47), the tinnitus group was divided based on individual TQ scores. When subdividing the tinnitus group, the regression analysis revealed that all three groups differed significantly on all SGI subscales except on the OI subscale, and that the control group and the compensated tinnitus group (TQ <47) did not differ on the D subscale (Fig. 1A).

Figure 1A illustrates the results for the SGI subscales. However, SGI question items can also be differentiated by specific modalities (auditory, visual, focus). When doing so, a clear difference between the groups was observed. In the auditory modality, all groups differed significantly from each other. However, in the visual modality and in the general focus questions, the compensated tinnitus group did not differ from the control group. Furthermore, the decompensated group showed significantly higher scores in all modalities compared with both the control group and the compensated tinnitus group (Fig. 1B).

### Analyses Within the Tinnitus Group

**Fig. 2.**
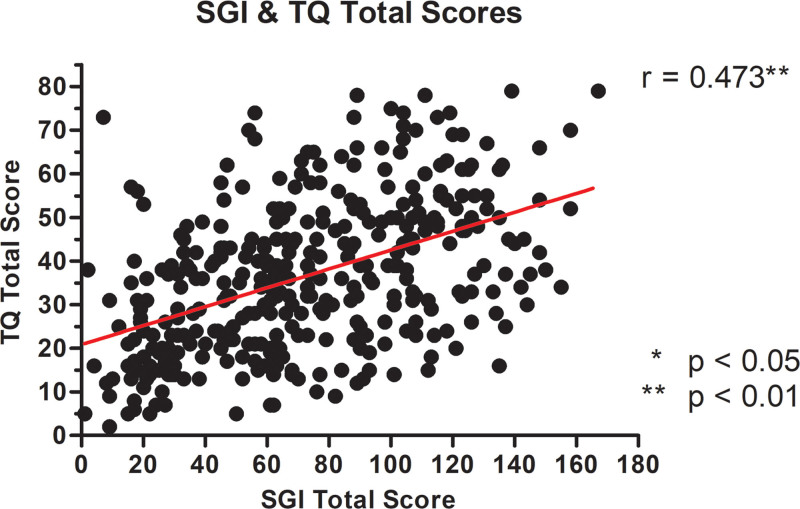
Scatterplot of the TQ and SGI total scores. SGI indicates Sensory Gating Inventory; TQ, tinnitus questionnaire.

A moderate (Akoglu 2018) correlation (*r* = 0.473) was observed between the SGI total score and the TQ total score (Fig. 2).

When analyzing the relation between SGI total score and TQ total score, we observed a significant relation (*B* = 0.146; 95% CI, 0.104 to 0.187; *p* < 0.001) when controlling for sex, tinnitus loudness, and tinnitus duration.

## DISCUSSION

As hypothesized, a significant positive correlation was observed between tinnitus severity and SG. The tinnitus group as a whole (combined compensated and decompensated) had significantly higher SGI scores than the control group. All three groups differed significantly on all SGI subscales except for the OI subscale, and the control group and the compensated tinnitus group (TQ <47) did not differ on the D subscale. Finally, in the auditory modality, all groups differed significantly from each other. For the other modalities (visual and focus), the compensated tinnitus group did not differ from the control group, and the decompensated group scored significantly higher compared with both groups.

The tinnitus group in the present study was slightly older (M = 54 y) and included fewer male persons (44.4%) than expected for the general Dutch population (M = 49 y; 57.2%) (Schubert et al. 2021; Centraal Bureau voor de Statistiek 2023). However, the amount of reported hearing loss (42%) was similar to previous reports (44.3%) (Schubert et al. 2021).

When subdividing the tinnitus group based on severity (compensated versus decompensated) and comparing the respective subgroups with each other and with the control group, some additional differences were observed. As expected, the compensated tinnitus group was more similar to the control group than the decompensated group. All three groups differed significantly on the total SGI score, while SG was most affected in the decompensated tinnitus group. Previous research that compared the SGI and the TQ (Mohebbi et al. 2019a) already confirmed significant differences between controls, compensated, and decompensated tinnitus groups across all subscales. Yet, the present study shows that these differences are not necessarily significant for all subscales. Most noticeably, there was no significant difference between the groups on the OI subscale. This is particularly noteworthy as this subscale includes items concerning the auditory modality. These OI items are all related to sound sensitivity and similar to questions from the Hyperacusis Questionnaire (Khalfa et al. 2002). They may therefore potentially fit better with symptoms of hyperacusis or hypersensitivity to sound rather than tinnitus. In addition, the decompensated tinnitus group scored significantly higher than the control group as well as the compensated tinnitus group on the D subscale, whereas the control group and the compensated group did not differ significantly. This suggests that decompensation interferes with attention capacity, as has been shown by previous research (Jacobson et al. 1996; Stevens et al. 2007; Lima et al. 2020). On the subscales PM and FS, all groups differed significantly. The PM subscale includes more auditory than visual items which could at least in part explain this difference in this subscale, since persons experiencing tinnitus are more focused on sounds in general. Concerning the FS subscale, it is known that stress and fatigue have an impact on tinnitus, stress symptoms, and exhaustion are directly related to tinnitus annoyance (Ciminelli et al. 2018). Therefore, a difference in FS between the subgroups can be expected.

When inspecting the different modalities of the SGI, a difference between all groups in the auditory modality was observed, indicating an increasing impairment in the auditory modality for each group. It is interesting that for the visual and focus modality, the control group and the compensated tinnitus group did not differ significantly. The decompensated tinnitus group, on the other hand, did differ significantly from the other two groups, suggesting that when tinnitus severity is high, other modalities are affected as well. This supports the observation that, for example, sustained and executive attention processes can be affected in tinnitus compared with control participants (Leong et al. 2020).

In previous research, Mohebbi et al. (2019a) suggested using only the auditory items of the SGI in tinnitus research. In this case, a significant difference between all three groups was observed in the auditory modality. It is interesting that in the visual and in the focus modality, the decompensated tinnitus group had significantly higher scores than both other groups. The control group and the decompensated group did not differ in these modalities (Fig. 1B). This could indicate that the decompensated tinnitus group experiences a more generalized SG impairment, whereas the impairment in the compensated group is primarily limited to the auditory modality.

As tinnitus is a very heterogeneous disorder (Cederroth et al. 2019), there are no clear neurophysiological measurements that can definitely be linked to it (Dornhoffer et al. 2006; Campbell et al. 2018; Brinkmann et al. 2024; Morse & Van der Werff 2024; Morse et al. 2024); however, there is potential in correlating the SGI to electrophysiological data. It has been shown previously that tinnitus severity can be correlated with neurophysiological measurement. Here, we showed that the SGI and TQ can be correlated; in turn, it can be expected that the SGI will also be related to neurophysiological data (Campbell et al. 2018, 2019). This can also be observed in the significant differences between the groups on the total SGI score. In addition, electrophysiology in persons with tinnitus has been related to poorer auditory gating which may be related to the observed differences on the auditory modality subscale. The electrophysiological findings concerning attention in tinnitus (Dornhoffer et al. 2006; Brinkmann et al. 2024) could be related to the differences in the focus modality subscale and the D subscale. In future research, there is an opportunity to compare these questionnaires to neurophysiological data (Brinkmann et al. 2024), potentially helping us identify a neural correlate for tinnitus. This step toward a more objective measure of tinnitus could be of interest for future research.

## CONCLUSION

The current findings confirm that SG is impaired in the PM, D, and OI domains as well as in the overall auditory modality in persons with tinnitus. We observed that this impairment was more prevalent in the decompensated tinnitus group, while results in the compensated tinnitus group were more similar to those obtained in the control group, except for the auditory modality. This pattern suggests that decompensated tinnitus entails a broader dysregulation than compensated tinnitus. Critically, the SGI results systematically covaried with tinnitus severity as measured by the TQ. It would therefore be interesting to investigate SG in the other affected modalities in decompensated tinnitus more closely with more specific tests for each subscale. This could inform about the transitional period and the processes that dysregulate when compensated tinnitus turns into decompensated tinnitus.

## ACKNOWLEDGMENTS

The ethical committee of Maastricht University approved the research project under the number ERCPN-OZL_233_06_02_2021_A1.
